# Genomic Profiling of Biliary Tract Cancers: Comprehensive Assessment of Anatomic and Geographic Heterogeneity, Co‐Alterations and Outcomes

**DOI:** 10.1002/jso.28081

**Published:** 2025-01-13

**Authors:** Diamantis I. Tsilimigras, Hunter Stecko, Dimitrios Moris, Timothy M. Pawlik

**Affiliations:** ^1^ Department of Surgery Division of Surgical Oncology, The Ohio State University Wexner Medical Center and James Comprehensive Cancer Center Columbus Ohio USA; ^2^ Department of Surgery, Duke University Hospital Duke University Durham North Carolina USA

**Keywords:** biliary, BTC, cholangiocarcinoma, co‐mutations, gallbladder cancer, genomic profiling, next‐generation sequencing

## Abstract

**Background:**

Biliary tract cancers (BTCs) represent distinct biological and genomic entities. Anatomic and geographic heterogeneity in genomic profiling of BTC subtypes, genomic co‐alterations, and their impact on long‐term outcomes are not well defined.

**Methods:**

Genomic data to characterize alterations among patients with BTCs were derived from the AACR GENIE registry (v15.1) and other genomic data sets. Patterns of mutational co‐occurrence, frequency of co‐alterations, and their impact on long‐term outcomes among BTC patients were examined.

**Results:**

Alterations in *IDH1* and *FGFR2* genes were mostly noted among intrahepatic cholangiocarcinoma (iCCA) samples, *TP53*, *ERBB2/HER2*, and *SMAD4* mutations were more frequent among gallbladder cancer (GBC) samples while extrahepatic cholangiocarcinoma (eCCA) more commonly harbored *KRAS* mutations (all *Q* < 0.001). Alterations in *IDH1* and *FGFR2* genes were more frequent among iCCA samples from Western vs. Eastern populations, while *KRAS*, *SMAD4*, and *ERBB2* mutations were more commonly observed among Eastern populations(all *Q* < 0.05). *FGFR2* gene was frequently co‐mutated with *BAP1* (log2OR: 1.55, *Q* < 0.001), while *IDH1* gene was commonly co‐mutated with *PBRM1* (log2OR: 1.09, *Q* < 0.001). Co‐alteration rate among patients with *IDH1*‐mutant iCCAs, *FGFR2*‐rearranged iCCAs, *KRAS*‐mutant eCCA, and *HER2*‐mutant GBCs were 80.8%, 85.2%, 76.7%, and 100%, respectively. Among patients with iCCA and *FGFR2* fusions/rearrangements, harboring co‐alterations in the *TP53* pathway or *PI3K* pathway correlated with worse overall survival (OS), while patients with *IDH1*‐mutant iCCA had worse OS when harboring co‐alterations in the cell cycle pathway.

**Conclusions:**

Marked genomic heterogeneity exists among patients with BTCs based on anatomic and geographic location. The overwhelming majority of BTC patients with clinically significant mutations had concurrent genomic co‐alterations. The current study highlights the molecular complexity of BTCs with multiple alterations that commonly co‐exist and could potentially be targeted to treat BTCs.

## 1 Introduction

Biliary tract cancers (BTCs) represent an aggressive category of malignancies that arise from the biliary epithelium [1]. Depending on their location in the biliary tree or gallbladder, BTCs can be subclassified as intrahepatic cholangiocarcinoma (iCCA), extrahepatic cholangiocarcinoma (eCCA) (which can be further subdivided into perihilar and distal cholangiocarcinoma), and gallbladder cancer (GBC) [2]. Due to the insidious onset of the disease, many patients with BTCs are diagnosed at an advanced stage when surgical resection is not an option [2, 3]. For patients with unresectable or metastatic disease at presentation, current National Comprehensive Cancer Network (NCCN) guidelines recommend gemcitabine plus cisplatin (GemCis) with the addition of durvalumab or pembrolizumab as first‐line therapy [4, 5], while patients who progress on these schemas usually receive fluorouracil, leucovorin, and oxaliplatin (FOLFOX) as second‐line therapy [6]. Nevertheless, response rates are generally in the range of 10%–30% with a median OS of less than 1 year [6]. As such, there has been an increased focus on identifying potentially actionable genetic alterations that may serve as targets to tailor therapy based on specific molecular profiles among patients with BTCs.

Although BTCs have largely been grouped together in clinical trials and treatment algorithms [5], iCCAs, eCCAs, and GBCs represent biological and genomic entities that are distinct from each other [7]. For example, genomic sequencing has demonstrated that iCCAs frequently harbor *IDH1/2* mutations and *FGFR2* fusions, whereas eCCAs and GBCs more commonly carry *KRAS* and *HER2* mutations, respectively [8, 9]. To date, most studies have been limited to a single center and have only analyzed a relatively low number of samples [8, 9]. In addition, while the etiology and prognosis of patients with BTCs may vary depending on geographical location (i.e., West vs. East) [1], differences in the genomic profile of BTCs from patients residing in Western vs. Eastern countries have not been well studied to date.

Genomic analyses of other gastrointestinal malignancies have demonstrated that most tumors are likely to have more than one potentially actionable mutations [10, 11]. In turn, targeting only one pathway may explain, in part, the relatively modest response rates associated with targeted monotherapy treatment in the setting of BTCs. Despite the availability of next‐generation sequencing (NGS) and advanced molecular analysis techniques, evaluation of co‐alterations in the setting of BTCs harboring clinically significant mutations is lacking. In addition, whether certain co‐alterations/mutations of BTCs (i.e., *IDH1*‐mutant iCCA, iCCA with *FGFR2* fusions/rearrangements, etc.) contribute to disparate outcomes remains unknown. Therefore, the objective of the current study was to assess the genomic heterogeneity of BTCs based on anatomic (i.e., iCCA, eCCA, GBC), as well as geographic location (i.e., West vs. East). In addition, we sought to characterize genomic co‐alterations among individuals with clinically significant mutations of BTCs, as well as define the impact on long‐term outcomes.

## 2 Methods

### 2.1 Genomic Profiling and Co‐Alterations of BTCs From American Association for Cancer Research (AACR) Genomics Evidence of Neoplasia Information Exchange (GENIE) Data Set

Data to characterize genomic alterations among patients with BTCs were derived from the American Association for Cancer Research (AACR) Genomics Evidence of Neoplasia Information Exchange (GENIE) data set v15.1 through cBioPortal [12]. The GENIE database was initially launched in 2017 and currently includes somatic genomic data from approximately 170 000 patients from 19 participating institutions [12]. All institutions employ NGS assays to detect somatic mutations, structural variants (e.g., fusions), and copy number alterations (e.g., amplifications or deletions) in formalin‐fixed paraffin‐embedded tumor samples using targeted panels of varying gene numbers [12]. Adult patients diagnosed with BTCs with available NGS data were identified and included in the analytic cohort. Data from the OncoKB database, provided by cBioPortal, were utilized to determine pathogenic gene alterations, as previously described [13, 14]. Germline mutations were excluded from the analysis. Genomic variations based on the anatomic location of BTCs (i.e., GBC, iCCA, and eCCA), as well as genomic co‐alterations involving important oncogenic pathways for each BTC subtype were examined.

### 2.2 Genomic Data From China Pan‐Cancer Data Set

An additional pan‐cancer database from China [15], available through cBioPortal (https://www.cbioportal.org/study/summary?id=pan_origimed_2020), was utilized to identify patients with BTCs with available NGS data. Differences in genomic profiling between Western (i.e., patients in GENIE database) and Eastern populations (i.e., Chinese pan‐cancer database) with different subtypes of BTCs were evaluated.

### 2.3 Genomic and Survival Data From the Memorial Sloan Kettering Cancer Center

Another large clinical‐genomic pan‐cancer database [16] from the Memorial Sloan Kettering Cancer Center (MSKCC) was utilized to assess the impact of certain genomic profiles of BTCs on long‐term outcomes. Because all data originated from publicly available de‐identified databases, the present study was deemed exempt from the Institutional Review Board of the Ohio State University.

### 2.4 Statistical Analysis

Data were expressed as median (interquartile range [IQR]) and frequency (%) for continuous and categorical variables, respectively. Differences in categorical variables were assessed with the chi‐squared or Fisher exact tests, while differences in continuous variables were assessed with the use of the Kruskal–Wallis test. The prevalence of the most frequent genomic alterations among patients with BTCs was calculated after accounting for the number of samples tested for each gene (i.e., correct denominator). Differences in the prevalence of genomic alterations among patients with different types of BTCs were calculated. To adjust for multiple hypotheses across multiple gene pairs, *p* values were corrected by the Benjamini‐Hochberg method to determine false discovery rate‐corrected *Q* values. Patterns of co‐occurrence and mutual exclusivity for certain pairs of variant genes were evaluated utilizing the mutual exclusivity modules statistical method from cBioportal [17] calculating log2 odds ratio to assess the direction of associations. Triangled heatmaps were generated to visualize the genetic pair associations for each BTC type. Differences in OS were assessed with the Kaplan–Meier method and the log‐rank test. The level of statistical significance was assessed at *a* = 0.05. All statistical analyses were performed with the online cBioPortal platform along with the addition of JMP v17 (SAS Institute Inc., Cary, NC) statistical software.

## 3 Results

### 3.1 Characteristics of BTC Patients and Samples in the AACR GENIE Cohort

A total of 1857 patients who underwent genomic profiling of BTCs were identified in the AACR GENIE database. Median age at the time of tumor sequencing was 65 years (IQR: 57–72). Most patients were White (*n* = 1303, 70.2%) followed by Asian (*n* = 152, 8.2%) and Black (*n* = 118, 6.4%); a subset of individuals self‐identified as “other/unknown” (*n* = 284, 15.2%). The male‐to‐female ratio was almost 1:1 (females: *n* = 1014, 54.6%; males: *n* = 837, 45.1%, unknown sex: *n* = 5, 0.3%). The vast majority of patients (*n* = 1737, 93.5%) had one tumor sample sequenced (total number of samples: *n* = 1912). Most samples were derived from the primary tumor (*n* = 1223, 64.0%), while the remaining samples were obtained from metastatic tumors (*n* = 589, 30.8%) or otherwise unspecified samples (*n* = 100, 5.2%).

Most patients underwent sequencing for iCCA (*n* = 1068, 57.5%) followed by GBC (*n* = 558, 30.0%) and eCCA (*n* = 231, 12.5%). The median number of mutations per sample was 4 (IQR: 2–6) for iCCA, 4 (IQR: 2–7) for eCCA, and 6 (IQR: 3–10) for GBC (*p* < 0.001). The fraction of the genome altered among individuals with iCCA, eCCA, and GBC was 0.17 (IQR: 0.04–0.31), 0.07 (IQR: 0–0.2), and 0.09 (IQR: 0–0.29), respectively (*p* < 0.001) (Table 1).

**Table 1:** Characteristics of patients with BTCs with available NGS data in the AACR GENIE database.

	Extrahepatic cholangiocarcinoma (*n* = 231)	Gallbladder cancer (*n* = 558)	Intrahepatic cholangiocarcinoma (*n* = 1068)	*p*
Age	66 (57–72)	67 (58–73)	64 (55–71)	< 0.001
Sex				< 0.001
Male	136 (58.9%)	169 (30.3%)	532 (49.8%)	
Female	93 (40.3%)	338 (69.5%)	533 (49.9%)	
Unknown	2 (0.9%)	1 (0.2%)	3 (0.3%)	
Race				< 0.001
White	167 (72.3%)	368 (65.9%)	768 (71.9%)	
Asian	23 (10.0%)	39 (7.0%)	90 (8.4%)	
Black	9 (3.9%)	55 (9.9%)	54 (5.1%)	
Unknown/other	32 (13.8%)	96 (17.2%)	156 (14.6%)	
Ethnicity				< 0.001
Hispanic	11 (4.8%)	65 (11.6%)	62 (5.8%)	
Non‐Hispanic	187 (81.0%)	385 (69.0%)	840 (78.7%)	
Unknown	33 (14.2%)	108 (19.4%)	166 (15.5%)	
No samples/patient				0.233
1	213 (92.2%)	534 (95.7%)	990 (92.7%)	
≥ 2	18 (7.8%)	24 (4.3%)	78 (7.3%)	
Sample type^a^				< 0.001
Primary tumor	140 (60.1%)	266 (46.6%)	817 (73.7%)	
Metastasis	85 (36.5%)	249 (43.6%)	255 (23.0%)	
Unknown	8 (3.4%)	56 (9.8%)	36 (3.3%)	
Mutation count/sample	4 (2–7)	6 (3–10)	4 (2–6)	< 0.001
Fraction of genome altered	0.07 (0–0.2)	0.09 (0–0.29)	0.17 (0.04–0.31)	< 0.001

a Number of samples: eCCA: *n* = 233; GBC: *n* = 571; iCCA: *n* = 1108.

### 3.2 Genomic Heterogeneity of BTCs Based on Anatomic Location

A total of 1091 genes were screened across 1912 samples. The most commonly mutated genes among iCCA samples were *TP53* (20.5%), *IDH1* (19.9%), *BAP1* (18.7%), *FGFR2* (15.8%), and *ARID1A* (17.1%). *IDH1* mutations were all missense (100%) mutations with the overwhelming majority representing single nucleotide variations at R132 in exon 4 (99.0%). Although *FGFR2* point mutations were identified only in 5.1% of iCCA samples, the prevalence of *FGFR2* fusions/rearrangements was 12.5%. The most commonly mutated genes among eCCA samples included *TP53* (44.4%), *KRAS* (37.8%), *SMAD4* (14.7%), *ARID1A* (14.3%), and *CDKN2A* (11.2%), while the most frequent alterations among GBC samples were enriched in *TP53* (60.0%), *ARID1A* (19.4%), *SMAD4* (18.3%), *CDKN2A* (13.9%), and *ERBB2/HER2* (11.0%) genes.

Marked genomic variations were observed based on the anatomic location of BTCs. Notably, alterations in *IDH1* (iCCA: 19.9% vs. eCCA: 1.3% vs. GBC: 1.1%, *Q* < 0.001) and *FGFR2* (iCCA: 15.8% vs. eCCA: 1.3% vs. GBC: 2.3%, *Q* < 0.001) genes were almost exclusively observed among iCCA samples; *BAP1*, *PBRM1*, and *IDH2* mutations were also more commonly enriched in iCCA vs. eCCA or GBC samples (all *Q* < 0.001). In contrast, *TP53* (iCCA: 20.5% vs. eCCA: 44.4% vs. GBC: 60.0%), *ERBB2/HER2* (iCCA: 3.9% vs. eCCA: 8.6% vs. GBC: 11.1%), and *SMAD4* (iCCA: 3.8% vs. eCCA: 14.7% vs. GBC: 18.3%) mutations were more frequent among GBC samples, while eCCA more commonly harbored *KRAS* mutations compared with other BTC types (iCCA: 12.4% vs. eCCA: 37.8% vs. GBC: 9.6%) (all *Q* < 0.001). No differences in the prevalence of *ARID1A*, *CDKN2A*, *CDKN2B*, *KMT2D*, *BRAF*, *ATM*, *PIK3CA*, *TERT*, *KMT2C*, *NF1*, and *RASA1* were noted among iCCA, eCCA, and GBC samples (all *Q* > 0.05) (Table 2).

**Table 2:** Prevalence of top 20 mutated genes among patients with iCCA, eCCA, and GBC (% calculated at the sample level).

Gene	Cytoband	Intrahepatic cholangiocarcinoma	Extrahepatic cholangiocarcinoma	Gallbladder cancer	*Q* value
*TP53*	17p13.1	227 (20.51%)	103 (44.40%)	342 (60.00%)	**< 0.001**
*IDH1*	2q34	220 (19.86%)	3 (1.29%)	6 (1.05%)	**< 0.001**
*BAP1*	3p21.1	191 (18.69%)	5 (2.31%)	5 (0.98%)	**< 0.001**
*FGFR2*	10q26.13	175 (15.79%)	3 (1.29%)	13 (2.28%)	**< 0.001**
*ARID1A*	1p36.11	175 (17.14%)	31 (14.35%)	99 (19.41%)	0.791
*CDKN2A*	9p21.3	155 (14.03%)	26 (11.21%)	79 (13.88%)	0.880
*KRAS*	12p12.1	137 (12.36%)	88 (37.77%)	55 (9.63%)	**< 0.001**
*PBRM1*	3p21.1	131 (13.56%)	9 (4.35%)	26 (5.23%)	**< 0.001**
*CDKN2B*	9p21.3	124 (12.14%)	15 (6.94%)	58 (11.46%)	0.514
*IDH2*	15q26.1	63 (5.80%)	0 (0.00%)	3 (0.55%)	**< 0.001**
*KMT2D*	12q13.12	62 (6.40%)	13 (6.28%)	43 (8.63%)	0.800
*BRAF*	7q34	60 (5.42%)	7 (3.00%)	16 (2.80%)	0.286
*ATM*	11q22.3	57 (5.25%)	16 (6.93%)	41 (7.54%)	0.701
*PIK3CA*	3q26.32	54 (4.87%)	10 (4.29%)	49 (8.58%)	0.104
*TERT*	5p15.33	51 (5.31%)	4 (1.94%)	32 (6.54%)	0.383
*KMT2C*	7q36.1	49 (6.09%)	9 (5.06%)	37 (10.16%)	0.272
*NF1*	17q11.2	47 (4.50%)	13 (5.88%)	29 (5.63%)	0.880
*ERBB2*	17q12	43 (3.88%)	20 (8.58%)	63 (11.05%)	**< 0.001**
*RASA1*	5q14.3	43 (5.08%)	5 (2.60%)	12 (2.78%)	0.497
*SMAD4*	18q21.2	42 (3.80%)	34 (14.66%)	104 (18.28%)	**< 0.001**

*Note:* Bold values denote statistical significance.

### 3.3 Genomic Heterogeneity of BTCs Based on Geographic Location

A subsequent analysis was conducted to examine genomic variations of BTCs based on geographic location. A Chinese pan‐cancer database [15] was interrogated to identify individuals with BTCs who had undergone NGS (Eastern cohort), which was compared with individuals with BTCs from the AACR GENIE database (Western cohort). *TP53* and *TERT* mutations were more frequent among samples from Eastern vs. Western populations across all BTC types (*TP53* mutations: iCCA: 43.1% vs. 20.5%; eCCA: 61.0% vs. 44.2%; GBC: 73.7% vs. 59.9%, all *Q* < 0.001; *TERT* mutations: iCCA: 15.9% vs. 4.6%; eCCA: 6.8% vs. 1.7%; GBC: 18.7% vs. 5.6%, all *Q* < 0.001). Alterations in *IDH1* and *FGFR2* genes were approximately three and two times more frequent among iCCA samples from Western vs. Eastern populations, respectively (Western vs. Eastern: *IDH1*: 19.9% vs. 6.7%; *FGFR2*: 15.8% vs. 7.4%, both *Q *< 0.001); *BAP1*, *FGFR2*, *CDKN2B*, *IDH2*, and *RASA1* mutations were also more frequent among iCCA samples from Western vs. Eastern cohorts (all *Q* < 0.001) (Table 3).

**Table 3:** Differences in prevalence of top 20 mutated genes among Western vs. Eastern patients with BTCs (% calculated at the sample level).

	Intrahepatic cholangiocarcinoma			Extrahepatic cholangiocarcinoma			Gallbladder cancer		
Gene	West (*n* = 1108)	East (*n* = 555)	*Q* value	West (*n* = 233)	East (*n* = 351)	*Q* value	West (*n* = 571)	East (*n* = 240)	*Q* value
*TP53*	20.5%	43.1%	**< 0.001**	44.2%	61.0%	**< 0.001**	59.9%	73.7%	**< 0.001**
*IDH1*	19.9%	6.7%	**< 0.001**	1.3%	1.1%	0.872	1.0%	0.4%	0.373
*BAP1*	17.2%	11.2%	**0.001**	2.2%	4.3%	0.166	0.9%	2.1%	0.155
*FGFR2*	15.8%	7.4%	**< 0.001**	1.3%	3.1%	0.153	2.3%	1.2%	0.337
*ARID1A*	15.8%	18.4%	0.182	13.3%	15.9%	0.378	17.3%	14.2%	0.265
*CDKN2A*	14.0%	12.2%	0.327	11.2%	16.8%	0.058	13.8%	22.5%	**0.002**
*KRAS*	12.4%	27.7%	**< 0.001**	37.8%	35.9%	0.645	9.6%	12.1%	0.295
*CDKN2B*	11.2%	4.0%	**< 0.001**	6.4%	2.3%	**0.011**	10.2%	7.1%	0.167
*PBRM1*	11.8%	11.9%	0.967	3.9%	4.3%	0.806	4.5%	4.2%	0.807
*KMT2D*	5.6%	7.4%	0.153	5.6%	6.8%	0.541	7.5%	11.7%	0.057
*BRAF*	5.4%	4.9%	0.634	3.0%	6.3%	0.075	2.8%	2.9%	0.928
*IDH2*	5.7%	1.8%	**< 0.001**	0.0%	0.3%	0.415	0.5%	0.4%	0.840
*ATM*	5.1%	5.2%	0.944	6.9%	4.3%	0.171	7.2%	7.5%	0.873
*PIK3CA*	4.9%	7.9%	**0.013**	4.3%	4.6%	0.878	8.6%	12.9%	0.059
*TERT*	4.6%	15.9%	**< 0.001**	1.7%	6.8%	**0.004**	5.6%	18.7%	**< 0.001**
*KMT2C*	4.4%	6.3%	0.098	3.9%	9.7%	**0.008**	6.5%	10.4%	0.054
*NF1*	4.2%	5.4%	0.287	5.6%	6.8%	0.541	5.1%	7.5%	0.178
*RASA1*	3.9%	0.0%	**< 0.001**	2.1%	0.0%	**0.005**	2.1%	0.0%	**0.024**
*SMAD4*	3.8%	9.2%	**< 0.001**	14.6%	21.6%	**0.032**	18.2%	14.6%	0.210
*ERBB2*	3.9%	3.8%	0.923	8.6%	6.3%	0.289	11.0%	18.8%	**0.003**

*Note:* Bold values denote statistical significance.

In contrast, KRAS mutations were more commonly observed in iCCA samples from Eastern populations (Western vs. Eastern: KRAS: 12.4% vs. 27.7%, *Q* < 0.001). Both iCCA and eCCA samples from Eastern populations also more frequently harbored mutations in *the SMAD4* gene (Western vs. Eastern; iCCA: 3.8% vs. 9.2%; eCCA: 14.6% vs. 21.6%, both *Q* < 0.05). Similarly, *ERBB2/HER2* mutations were more often identified among GBC samples from Eastern (18.8%) vs. Western populations (11.0%) (*Q* = 0.003) (Table 3).

### 3.4 Patterns of Co‐Occurrence, Mutual Exclusivity, and Co‐Alterations Among Patients With BTCs

**Figure 1:**
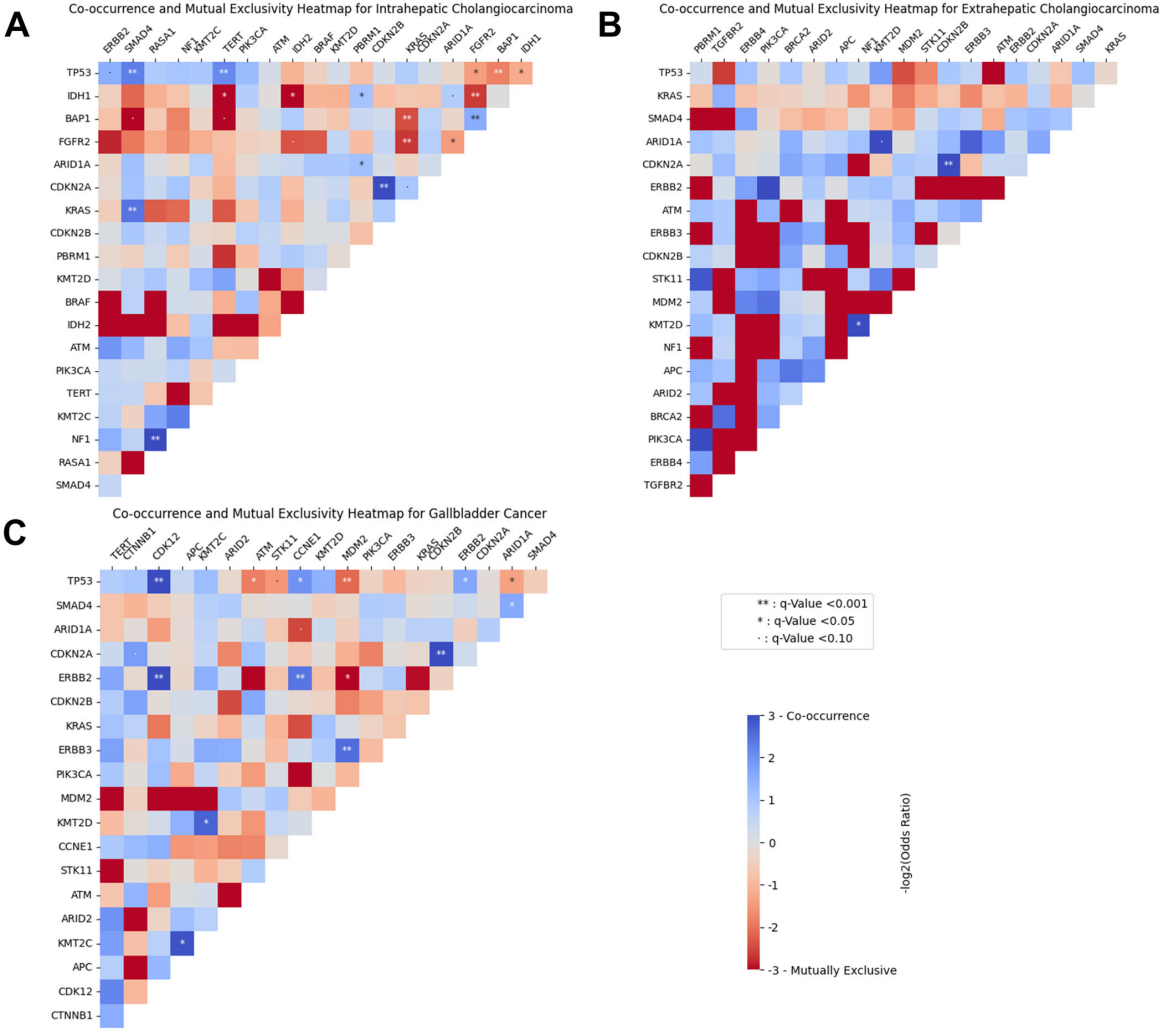
Patterns of co‐occurrence and mutual exclusivity in genomic alterations among patients with iCCA (A), eCCA (B), and GBC (C).

Evaluation of co‐occurrence and mutual exclusivity patterns of genomic pairs among individuals with BTCs from the AACR GENIE cohort was then performed. Notably, among patients with iCCA, *IDH1* and *FGFR2* alterations were mutually exclusive (log2OR: −2.70, *Q* < 0.001); the *FGFR2* gene was frequently co‐mutated with *BAP1* (log2OR: 1.55, *Q* < 0.001), while the *IDH1* gene was commonly co‐mutated with *PBRM1* (log2OR: 1.09, *Q* < 0.001) (Figure 1A). Among patients with eCCA, *CDKN2A* and *CDKN2B* alterations co‐occurred (log2OR: > 3, *Q* < 0.001), as did alterations in *KMT2D* and *NF1* genes (log2OR: > 3, *Q* < 0.001) (Figure 1B). Notably, *ERBB2/HER2* alterations among GBC patients co‐occurred with alterations in *CDK12* (log2OR: > 3) and *CCNE1* (log2OR: 2.43) (i.e., cell cycle pathway) and *TP53* genes (both *Q* < 0.05, Figure 1C).

**Figure 2:**
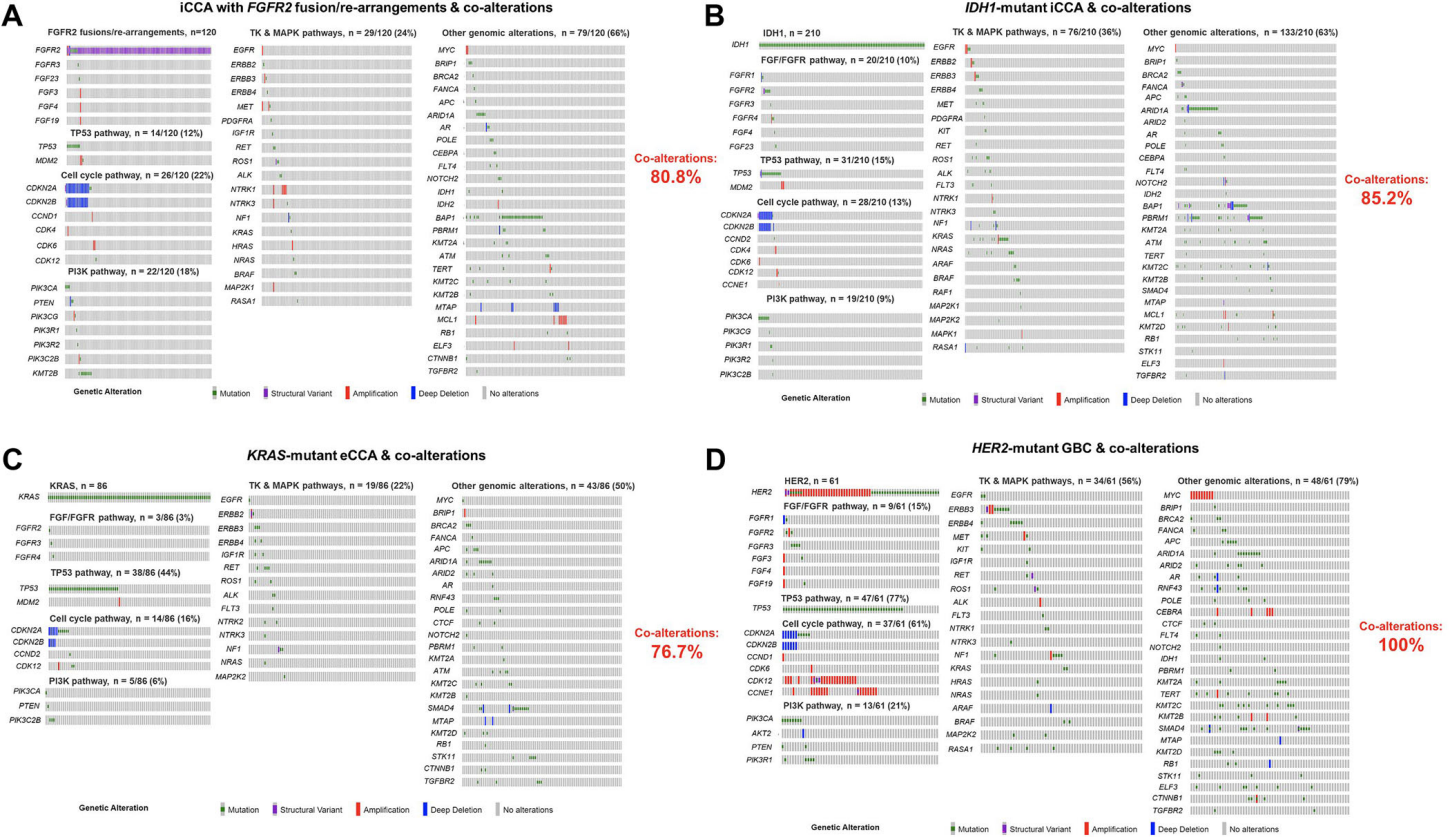
Oncoprints demonstrating co‐alterations among patients who had iCCA with *FGFR2* fusions/rearrangements (A) and iCCA with *IDH1* mutations (B). Oncoprints demonstrating co‐alterations among patients who had *KRAS*‐mutant eCCA (C) and *HER2‐* mutant GBC (D).

The prevalence of co‐alterations among individuals with clinically significant mutations of BTCs was then assessed. Patients with *FGFR2*‐rearranged iCCA (*n* = 120) had co‐alterations in 80.8% of cases; the most frequently co‐altered pathways/genes were *RTK‐MAPK* pathways (24%), cell cycle pathways (22%), and other genomic alterations (most importantly *BAP1* mutations) (66%) (Figure 2A). In addition, individuals with *IDH1*‐mutant iCCA (*n* = 210) had co‐alterations in 85.2% of cases with the most frequently altered pathways being the *RTK‐MAPK* pathways (36%), and other co‐alterations (most importantly *ARID1A*, *BAP1*, and *PBRM1* mutations) (63%) (Figure 2B). The prevalence of genomic co‐alterations among individuals with *KRAS*‐mutated eCCA and *HER2*‐mutant GBC was 76.7% and 100%, respectively (Figure 2C,D).

### 3.5 Outcomes of Patients With *IDH1*‐Mutant and *FGFR2*‐Rearranged ICCAs With Co‐Alterations

**Figure 3:**
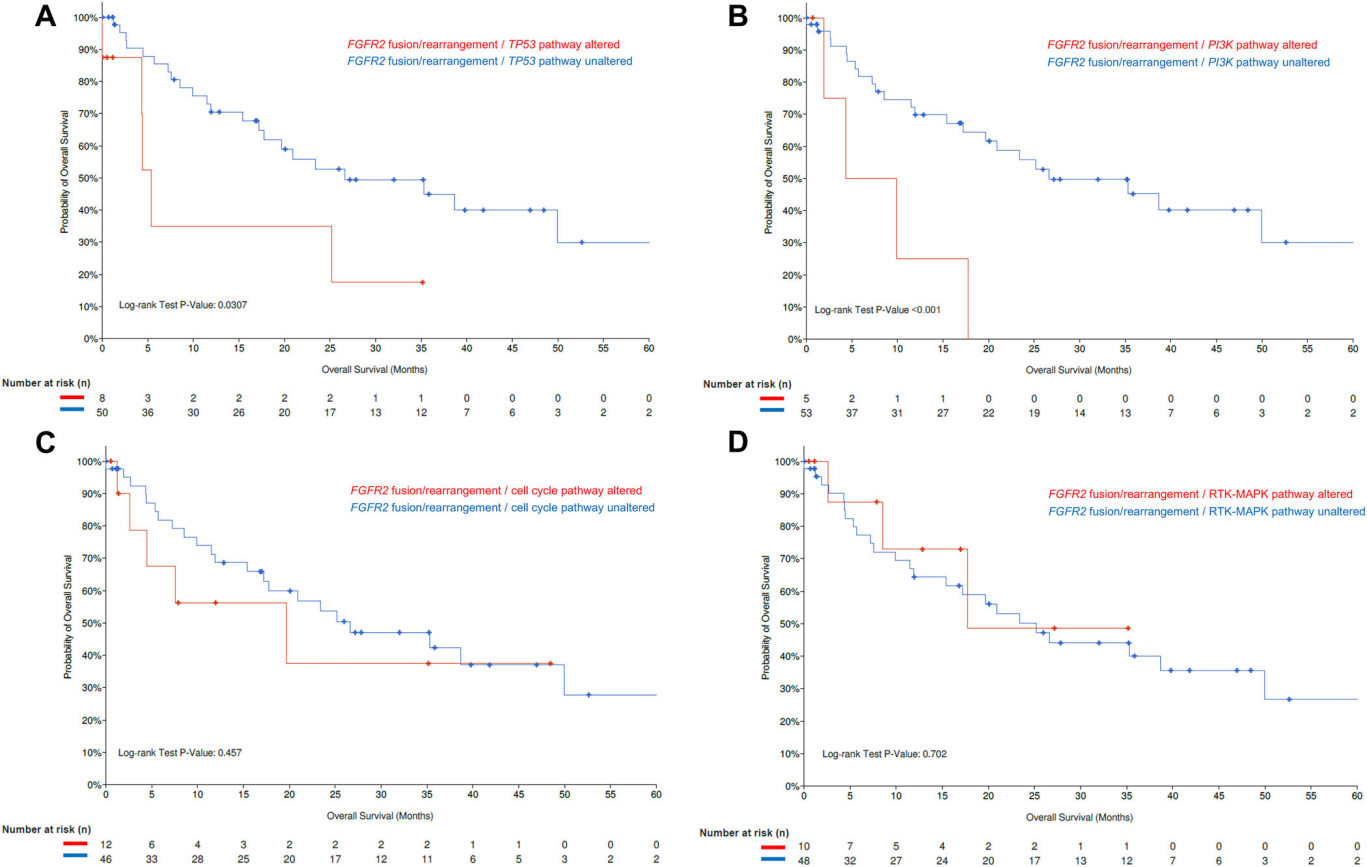
Kaplan–Meier curves demonstrating differences in OS among patients with iCCA with *FGFR2* fusions/rearrangements and *TP53* pathway (A), *PI3K* pathway (B), cell cycle pathway (C), and *RTK‐MAPK* alterations (D).

**Figure 4:**
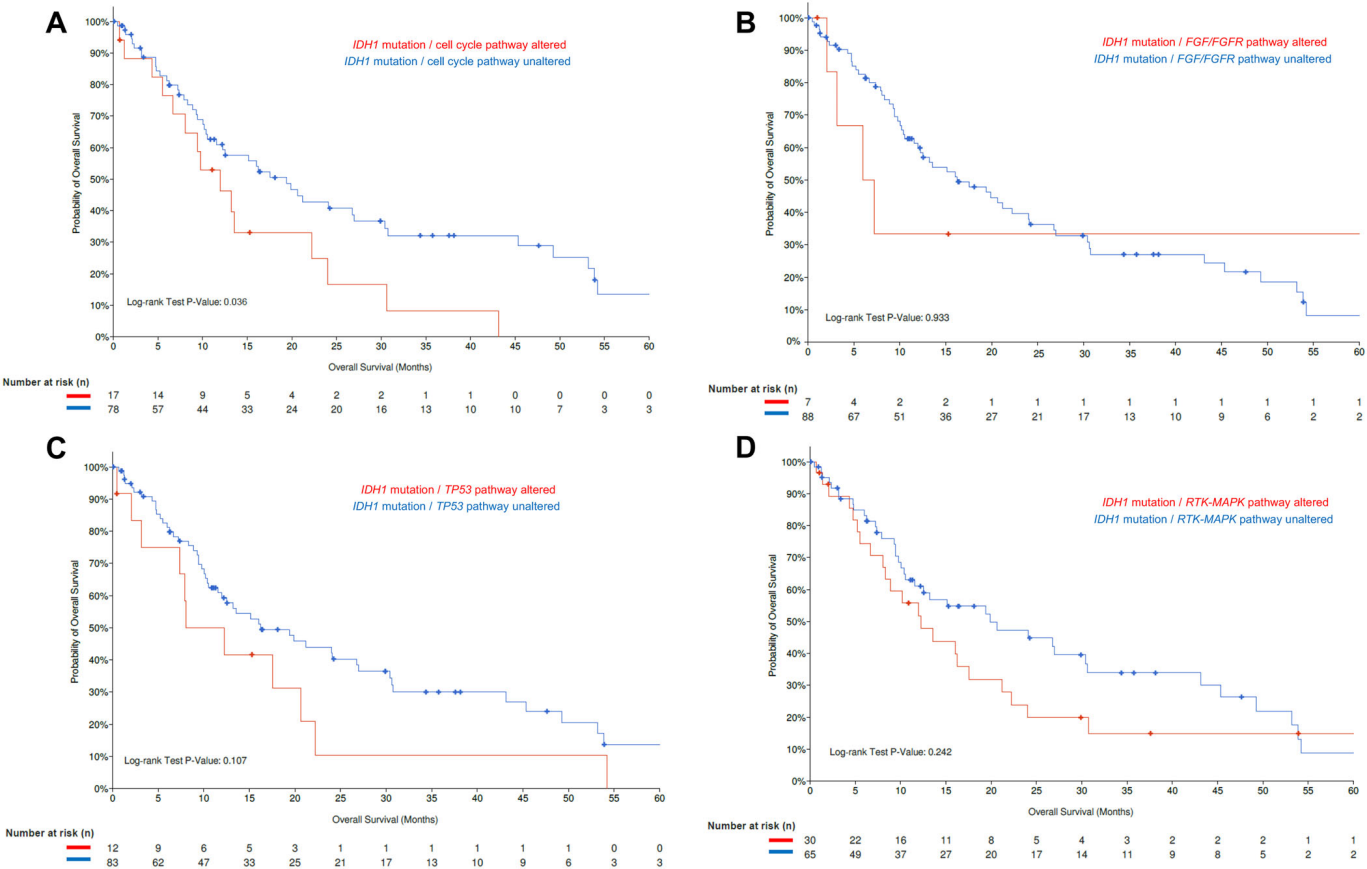
Kaplan–Meier curves demonstrating differences in OS among patients with *IDH1*‐mutant iCCA and cell cycle pathway (A), *FGF/FGFR* pathway (B), *TP53* pathway (C), and *RTK‐MAPK* alterations (D).

The MSKCC MetTropism database [16] (Table S1) was further interrogated to identify individuals with *IDH1*‐mutant (*n* = 95 out of 407 iCCA samples), as well as *FGFR2*‐rearranged iCCA (*n* = 58 out of 407 iCCA samples). Patients with *FGFR2*‐rearranged iCCA harboring co‐alterations in the TP53 pathway or the PI3K pathway had worse OS compared with iCCA patients with *FGFR2* fusions/rearrangements without these co‐alterations (*FGFR2* fusion/*TP53* pathway altered vs. *FGFR2* fusion/*TP53* pathway unaltered; median OS: 5.4 [95% CI: 4.3‐NA] vs. 26.6 [95% CI: 17.8‐NA] months, *p* = 0.031; *FGFR2* fusion/*PI3K* pathway altered vs. *FGFR2* fusion/*PI3K* pathway unaltered; median OS: 4.3 [95% CI: 1.9‐NA] vs. 26.6 [19.7‐NA] months, *p* < 0.001) (Figure 3A,B). In contrast, patients with both FGFR2 alterations and co‐alterations in cell cycle or *RTK‐MAPK* pathways had comparable outcomes with patients who did not have these co‐altered pathways (*FGFR2* fusion/cell cycle pathway altered vs. *FGFR2* fusion/cell cycle pathway unaltered; median OS: 19.7 [95% CI: 4.4‐NA] vs. 26.6 [95% CI: 17.2‐NA] months, *p* = 0.457; *FGFR2* fusion/*RTK‐MAPK* pathway altered vs. *FGFR2* fusion/*RTK‐MAPK* pathway unaltered; median OS: 17.8 [95%CI 8.5‐NA] vs. 25.2 [95%CI 15.4‐NA] months, *p* = 0.702, Figure 3C,D). Furthermore, patients with *IDH1*‐mutant iCCA and co‐alterations in the cell cycle pathway had shorter OS compared with individuals without these co‐alterations (median OS: 11.9 [95% CI: 8.08‐NA] vs. 19.4 [95% CI: 12.2–30.4] months, *p* = 0.036 (Figure 4A); co‐alterations in any other pathways were not associated with worse long‐term outcomes (Figure 4B–D).

## 4 Discussion

BTCs represent a heterogenous, and aggressive group of malignancies with a poor overall prognosis [1, 7]. The efficacy of currently available cytotoxic chemotherapy regimens to treat BTCs has been limited. In turn, there is increasing interest to develop molecular‐based therapies that could offer a better chance for long‐term survival [5]. Developing effective, targeted therapies requires comprehensive molecular analysis to better understand the molecular and genomic complexity of BTCs [7]. The current study was important because we comprehensively examined the genomic heterogeneity of BTCs based on their anatomic and geographic origin and evaluated the patterns of genomic co‐alterations, as well as the related prognostic implications. Notably, alterations in *IDH1* and *FGFR2* genes were almost exclusively observed in iCCA, whereas *KRAS* and *ERBB2*/*HER2* mutations were more commonly enriched in eCCA and GBC, respectively. While alterations in *TP53*, and *TERT* mutations were more common among Eastern populations across all types of BTCs, alterations in *IDH1* and *FGFR2* genes were approximately three and two times more frequent among iCCA in Western populations. In contrast, both *KRAS* and *SMAD4* mutations were more common among patients with iCCA from Eastern populations. The overwhelming majority of patients with clinically significant mutations had concurrent genomic co‐alterations; specifically, 80.8% of *IDH1*‐mutant iCCAs, 85.2% of iCCAs with *FGFR2* fusions, 76.7% of *KRAS*‐mutant eCCA and 100% of *HER2*‐mutant GBCs had other co‐alterations. Among patients who had iCCA with *FGFR2* fusions/rearrangements, harboring co‐alterations in the *TP53* pathway or *PI3K* pathway correlated with worse OS (vs. patients with FGFR2 fusions without these co‐signals), while patients with *IDH1*‐mutant iCCA had worse OS when harboring co‐alterations in the cell cycle pathway. To the best of our knowledge, this is the largest study to evaluate the genomic heterogeneity of BTCs and the first to examine co‐alterations and their impact on outcomes among patients with sequenced BTCs.

Previous research has demonstrated that iCCA, eCCA, and GBC are biologically distinct entities [7, 18]. In a comprehensive analysis of whole exome and transcriptome sequencing of 260 patients with BTCs, Nakamura et al. identified *IDH1* mutations in 20% of iCCA cases and *FGFR2* fusions in 14% of cases [7]. Prior genomic analyses reported by Jusakul et al. demonstrated *KRAS* mutations in 37.8% of eCCA cases and *ERBB2* amplifications in 11% of GBC cases [18]. These data were consistent with findings in the current study, which serve to highlight the varying prevalence of certain genetic mutations across BTC subtypes. Beyond the anatomic‐based variations in genomic profiles, the current study also demonstrated marked geographic variation in the genomic signatures of BTCs. While *TP53* and *TERT* mutations were more commonly observed in Eastern populations across all BTC subtypes, a higher prevalence of *IDH1*, *FGFR2*, and *BAP1* alterations were noted among Western populations. Although the reason behind these marked variations was likely multifactorial, differences in genomic profiles of Eastern vs. Western populations with BTCs might reflect differences in the etiology and pathogenesis of these diseases at different geographical locations [19, 20]. For example, in Southeast Asia, the liver fluke *Opisthorchis viverrini* is the leading cause of CCA, whereas in Western countries primary sclerosing cholangitis and nonalcoholic fatty liver disease (NAFLD) represent the main risk factors for the development of CCA [1]. Interestingly, prior research has demonstrated that *TP53* and *ARID1A* mutations are highly enriched in fluke‐related CCAs, whereas *BAP1* and *IDH1*/2 mutations are highly enriched in non‐fluke‐related CCAs [1]. These findings likely suggest potential regional environmental factors that influence trends related to different mutations. In turn, the data highlight the importance of considering geographic and ethnic backgrounds when developing targeted therapies for BTCs.

The current study provided a comprehensive assessment of co‐alterations in BTCs. Notably, the overwhelming majority of BTC patients with clinically significant mutations harbored other concurrent genomic alterations. Indeed, among individuals with *IDH1*‐mutant iCCA and *FGFR2*‐rearranged iCCA, 80.8% and 85.2% had other co‐alterations, respectively. To date, *IDH1*/2 inhibitors (i.e., ivosidenib) and *FGFR2* inhibitors (i.e., pemigatinib, futibatinib) have received FDA approval for the treatment of patients with advanced, refractory, metastatic cholangiocarcinoma with *IDH* mutations and *FGFR2* fusions/rearrangements, respectively, yet with limited response/duration of response [2, 5]. In the current study, we noted a high prevalence of concurrent alterations among iCCA individuals with *IDH1* mutations and *FGFR2* fusions/rearrangements suggesting that concurrent mutations might at least in part explain the non‐durable survival benefit associated with single‐agent targeted therapies.

Data from the current study also demonstrated that patients with *FGFR2*‐rearranged iCCA and co‐alterations in the *TP53* and *PI3K* pathways had worse OS compared with their counterparts. In particular, patients with *FGFR2* fusions and *TP53* pathway alterations had a median OS of 5.4 vs. 26.6 months among individuals without these co‐alterations. Similarly, *FGFR2* fusions with *PI3K* pathway alterations were associated with a median OS of 4.3 months. These findings align with previous studies indicating that *TP53* and *PI3K* pathway alterations are markers of aggressive disease and poor prognosis [1, 7]. In addition, patients with *IDH1*‐mutant iCCA with co‐alterations in the cell cycle pathway had a median OS of 11.9 vs. 19.4 months among patients without these co‐alterations. Collectively, the findings indicate the need to investigate the possibility of potential benefit with combination therapies targeting both *FGFR2* or *IDH1* and the co‐altered pathways, when possible, in an attempt to improve prognosis of patients with BTCs. The data support the findings of the I‐PREDICT trial, which suggested that combination targeted therapies for a variety of solid tumors are more likely to provide survival benefit compared with monotherapies [11]. This is particularly relevant for BTCs, given the high prevalence of co‐alterations observed in our study.

The current study is the largest analysis of patients with BTCs from multiple academic institutions worldwide and the first comprehensive evaluation of co‐alterations for each subtype of BTCs to date. Genomic data from the AACR GENIE database were aggregated from multiple academic centers each utilizing different NGS assays with different gene panel sets and tumor content criteria [12]. The use of cBioPortal platform allowed us to retrieve the actual number of patients tested for each gene (i.e., correct denominator), and calculate mutational frequency of certain genes accurately [17]. However, the genomic databases utilized in the context of this study lacked detailed information regarding patient‐level characteristics including comorbidities, obesity, smoking, etiology of disease. Thus, the association of these factors with certain genomic profiles among Western vs. Eastern populations could not be assessed. Given the lack of detailed information on cancer stage and treatment characteristics, the prognostic impact of these factors among BTC individuals with certain genomic signatures could also not be assessed. In addition, due to the limited number of patients with *KRAS*‐mutant eCCA and *HER2*‐mutant GBC, the impact of co‐alterations among patients with these tumors could not be determined. Future prospective studies will be required to validate our findings and investigate the therapeutic potential of combination targeted therapies in different BTC subtypes.

In conclusion, BTCs represent a diverse group of cancers characterized by marked genomic and geographic heterogeneity. The overwhelming majority of BTC patients with clinically significant mutations had concurrent genomic co‐alterations. Patients with *FGFR2* rearranged iCCA harboring co‐alterations in the *TP53* pathway or *PI3K* pathway had worse OS than patients with *FGFR2* fusions without these co‐signals, while patients with *IDH1*‐mutant iCCA had worse OS when harboring co‐alterations in the cell cycle pathway. The current study highlights the importance of considering geographic and ethnic backgrounds when developing targeted therapies for BTCs, as well as the importance of considering the molecular complexity of BTCs with multiple alterations that co‐exist and could potentially be targetable to treat BTCs.

## Conflicts of Interest

The authors declare no conflicts of interest.

## Synopsis

Biliary tract cancers (BTCs) displayed a marked genomic heterogeneity based on their anatomic and geographic location.

The overwhelming majority of BTC patients with clinically significant mutations had concurrent genomic co‐alterations highlighting the molecular complexity of BTCs with multiple alterations that commonly co‐exist and could be potentially targetable to treat BTC.

## Supporting information

Supporting information.

## Data Availability

The data that support the findings of this study are openly available from the CBioPortal platform (https://www.cbioportal.org/). AACR GENIE data are available online (https://genie.cbioportal.org/) upon request and acceptance by the AACR committee.
