# Supplementary material for: Genomic Profiling of Biliary Tract Cancers: Comprehensive Assessment of Anatomic and Geographic Heterogeneity, Co‐Alterations and Outcomes
Source: J Surg Oncol. 2025 Jan 13;131(7):1352–61. doi: 10.1002/jso.28081 (PMC12186109; doi:10.1002/jso.28081)
Supplement: Supplementary file 1 — Supporting information. [file JSO-131-1352-s001.docx]

**Supplemental Table 1** Characteristics of patients with BTCs in the MSKCC MetTropism database

|  | **Overall cohort**  **(n=697 patients)** |
| --- | --- |
| **Age at sequencing, yrs** | 65 (55-72) |
| **Sex** |  |
| **Male** | 328 (47.1%) |
| **Female** | 369 (52.9%) |
| **Unknown** | 0 (0%) |
| **Race** |  |
| **White** | 520 (74.6%) |
| **Asian** | 63 (9.0%) |
| **Black** | 44 (6.3%) |
| **Unknown/Other** | 70 (10.1%) |
| **No samples/patient** |  |
| **1** | 697 (100%) |
| **>2** | 0 (0%) |
| **Sample type** |  |
| **Primary tumor** | 470 (67.4%) |
| **Metastasis** | 227 (32.6%) |
| **Unknown** | 0 (0%) |
| **Cancer Type** |  |
| **iCCA** | 407 (58.4%) |
| **eCCA** | 119 (17.1%) |
| **GBC** | 171 (24.5%) |
| **Mutation Count/sample** | 3 (2-5) |
| **Fraction of genome altered** | 0.10 (0.01-0.25) |

iCCA: intrahepatic cholangiocarcinoma; eCCA: extrahepatic cholangiocarcinoma; GBC: gallbladder cancer
